# Discovering the Biological Significance and Therapeutic Potential of miR-29b-3p in Triple-Negative Breast Cancer

**DOI:** 10.3390/ijms24055048

**Published:** 2023-03-06

**Authors:** Ancuta Jurj, Oana Zanoaga, Lajos Raduly, Vlad Morhan, Zsofia Papi, Cristina Ciocan, Laura-Ancuta Pop, Ioana Berindan-Neagoe, Cornelia Braicu

**Affiliations:** 1 Research Center for Functional Genomics, Biomedicine and Translational Medicine, Iuliu Hatieganu University of Medicine and Pharmacy, 400347 Cluj-Napoca, Romania; 2 Faculty of Medicine, Iuliu Hațieganu University of Medicine and Pharmacy, 400347 Cluj-Napoca, Romania; 3 Faculty of Medicine, University of Szeged, 6720 Szeged, Hungary

**Keywords:** miR-29b-3p inhibitor, triple-negative breast cancer, miRNA, drug resistance

## Abstract

The lack of estrogen or progesterone receptors and absence of *HER2* amplification/overexpression in triple-negative breast cancer (TNBC) restricts therapeutic options used in clinical management. MicroRNAs (miRNAs) are small, non-coding transcripts which affect important cellular mechanisms by regulating gene expression at the post-transcriptional level. Among this class, attention was focused on miR-29b-3p with a high profile in TNBC and correlated with the overall survival rates, as TCGA data revealed. This study aims to investigate the implication of the miR-29b-3p inhibitor in TNBC cell lines by identifying a potential therapeutic transcript, improving the clinical outcomes of this disease. The experiments were performed on two TNBC cell lines (MDA-MB-231 and BT549) as in vitro models. An established dose of 50 nM was used for all functional assays performed on the miR-29b-3p inhibitor. A decreased level of miR-29b-3p determined a significant reduction in cell proliferation and colony-forming capacity. At the same time, the changes occurring at the molecular and cellular levels were highlighted. We observed that, when inhibiting the expression level of miR-29b-3p, processes such as apoptosis and autophagy were activated. Further, microarray data revealed that the miRNA expression pattern was altered after miR-29b-3p inhibition, pointing out 8 overexpressed and 11 downregulated miRNAs specific for BT549 cells and 33 upregulated and 10 downregulated miRNAs that were specific for MDA-MB-231 cells. As a common signature for both cell lines, three transcripts were observed, two downregulated, miR-29b-3p and miR-29a, and one upregulated, miR-1229-5p. According to DIANA miRPath, the main predicted targets are related to ECM (extracellular matrix) receptor interaction and TP53 signaling. An additional validation step through qRT-PCR was performed, which showed an upregulation of *MCL1* and *TGFB1*. By inhibiting the expression level of miR-29b-3p, it was shown that complex regulatory pathways targeted this transcript in TNBC cells.

## 1. Introduction

Breast cancer is a significant public health challenge. Despite sharing a common primary site, breast cancer is known to be a highly heterogeneous disease [1,2,3]. It opens new paths for oncology research to identify new therapeutic approaches to improve the clinical outcome of the diagnosed patients [4].

Breast cancer is generally classified according to the presence or absence of three receptors, the estrogen receptor (ER), progesterone receptor (PR) and human growth factor receptor 2 (HER2/neu) [5]. Triple-negative breast cancer (TNBC) represents 10–30% of all types of breast cancer and is characterized by the lack of all the above-mentioned receptors. TNBC is associated with poor clinical outcomes, being diagnosed in an advanced stage of disease, early relapse and metastasis in the brain and lung [6,7]. The standard therapeutic approaches for TNBC are represented by surgery, radiotherapy, chemotherapy, immunotherapy, targeted therapy and a combination of them [8]. To identify potential biomarkers and therapeutics for TNBC, it is necessary to provide a comprehensive understanding of the signaling pathways which govern this disease to establish new therapeutic approaches for TNBC patients [9].

MicroRNAs (miRNA) are endogenous, short (19 to 24 nucleotides), non-coding RNA strands with the function of sequence-specific gene expression regulation [10]. They acquire their gene silencing effect by binding to the 3′-UTR regions of specific mRNAs via partial complementary to their “seed” sequence and inhibiting the translation of the target mRNA [11]. miRNAs have an essential regulatory role in many biological processes, including cell proliferation, apoptosis, angiogenesis, drug resistance, invasion and metastasis. A miRNA’s expression level can be up- or downregulated in cancer cells compared to their normal counterparts, acting as oncomiRs or tumor suppressors [12]. Each miRNA can affect the expression of several different genes, and, in turn, one mRNA can be targeted by numerous other miRNA species [13]. This can be exploited as a therapeutic target for deciphering new mechanistic insights in pathological status, including TNBC.

The miR-29 family consists of three members: miR-29a, miR-29b and miR-29c [14], targeting several different regulatory pathways related to cancer hallmarks [15]. miR-29 was demonstrated to have an important role in various cancers, and it has been revealed to regulate multiple oncogenic processes. Although miR-29 has been comprehensively attested as a tumor suppressor in most studies, others have shown contradictory results [14,16].

This study aimed to investigate the cellular and molecular alterations induced as an effect of a miRNA inhibitor designed for silencing miR-29b-3p using antisense oligonucleotides, in parallel with a negative control inhibitor (a validated random sequence tested on mammalian cells and tissues, validated to have no effects on known miRNA function) [17,18] and a transient transfection protocol. To better understand these regulatory pathways, a microarray platform was used to identify the altered miRNA patterns, followed by the validation of the most relevant target genes, as well as the correlation with mutational signature and generation of miRNA-mRNA networks.

## 2. Results

### 2.1. The Expression Level of miR-29b-3p in TNBC Tumor Tissue and Cell Lines

**Figure 1:**
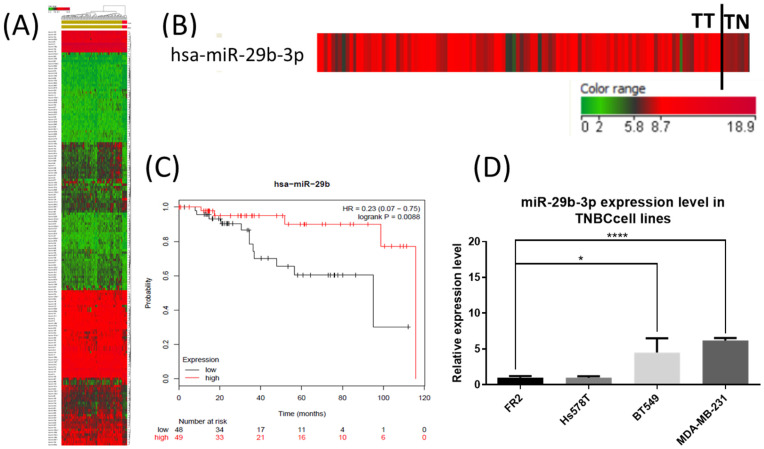
The expression level of miR-29b-3p in TNBC (TCGA dataset) and breast cancer cell lines. (**A**) The expression level of miR-29b-3p in TNBC versus adjacent normal tissue according to TCGA patient’s cohort. (**B**) A detailed view of miR-29b-3p expression level in TNBC patients according to TCGA patient’s cohort (TT: tumor tissue, TN: normal adjacent tissue). (**C**) The overall survival rate for miR-29b-3p in TNBC patients, according to data available from the online application KM Plotter developed based on TCGA data. Patients with expression above the median are indicated in the red line, and patients with expression below the median in the black line compared by the log-rank test, HR: hazard ratio (**D**) The evaluation of miR-29b-3p expression levels in triple-negative breast cancer cell lines (Hs578T, BT549 and MDA-MB-231), compared to the expression level for this transcript in normal epithelial breast cell line, FR2, based on ΔΔCt method and U6 for normalization (* *p* < 0.05, **** *p* < 0.0001).

By analyzing the miRNA dataset of TNBC from The Cancer Genome Atlas (TCGA), we found that the expression level of miR-29b-3p was upregulated in TNBC tissues compared to the normal adjacent tissues. In Figure 1A, we presented the miRNAs observed altered in TNBC compared to adjacent normal tissue. In Figure 1B, we represented the detailed expression level of miR-29b-3p in TNBC. In addition, the correlation of miR-29b-3p expression with the overall survival rate is shown in Figure 1C, showing KM Plotter graphical analysis based on TCGA data.

Further, we analyzed the expression level of miR-29b-3p by qRT-PCR in triple-negative breast cancer cell lines (Hs578T, BT549 and MDA-MB-231) and a normal breast epithelial cell line (FR-2). To evaluate the effect of miR-29b-3p transitory inhibition, the following TNBC cell lines were selected, BT549 and MDA-MB-23 cells, because of the significantly higher miR-29b-3p expression levels compared to the FR-2 cell line.

**Figure 2:**
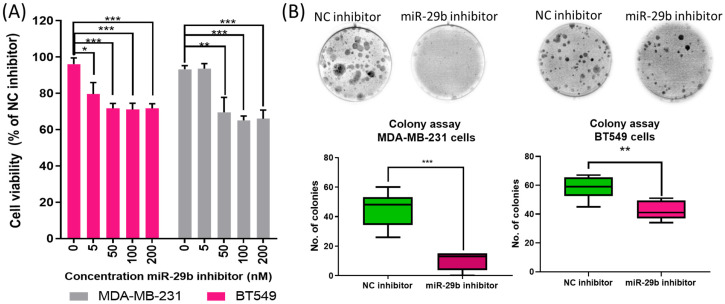
The inhibition of miR-29b-3p reduces cell viability and the capacity for colony formation. (**A**) Using MTT assay, it was observed that the downregulation of miR-29b-3p reduces cell viability rate in both TNBC cell lines (**B**). The inhibition of miR-29b-3p reduced the capacity of colony assay formation. Colony formation was observed in both TNBC cell lines transfected with miR-29b-3p inhibitor and NC (negative control) inhibitor. Data are presented as % of NC inhibitor group, NC is considered as 100%; data presented as mean ± SD, n = 3; Student’s *t*-test was considered statistically significant for * *p* < 0.05, ** *p* < 0.01, *** *p* < 0.001 when compared to the NC inhibitor group.

A preliminary screening test was conducted to evaluate the effect on cell viability by using different concentrations of miR-29b-3p inhibitor. An MTT assay showed a significant inhibition on cell viability, starting with 50 nM of miR-29b-3p, the concentration used for all the in vitro experiments (Figure 2A). Downregulation of miR-29b-3p reduces cell viability and the capacity of colony formation assay.

Further, a colony formation assay was used to evaluate cell proliferation ability (Figure 2B). In the case of the MDA-MB-231 cell line, the mean number of colonies was 45.00 ± 11.93 (average ± SD) for negative control inhibitor, respectively, 10.17 ± 6.24 for the transfected TNBC cells with miR-29b-3p. We obtained similar results for the BT549 cell line, 58.33 ± 7.89 colonies for the negative control inhibitor and 43.33 ± 6.62 colonies for the transfected TNBC cells with miR-29b-3p. These data showed a significant inhibition of colony formation in the case of TNBC cells transfected with miR-29b-3p inhibitor compared to the negative control inhibitor (Figure 2).

### 2.2. miR-29b-3p Inhibits Mitochondrial Activity and Activates Autophagy and Apoptosis

**Figure 3:**
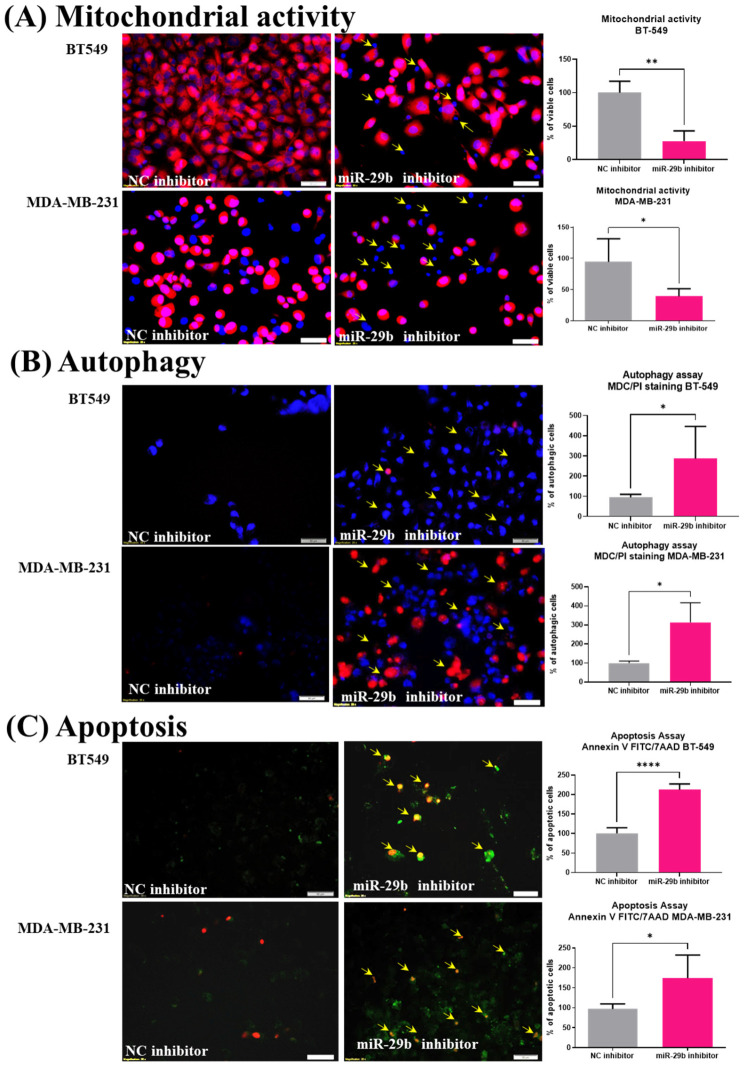
miR-29b-3p inhibits mitochondrial activity and activates autophagy and apoptosis in both TNBC cell lines. (**A**) The inhibition of miR-29b-3p reduces the mitochondrial activity in transfected TNBC cell lines, shown through TMRE/Hoechst double staining (active mitochondria are stained in red; cell nuclei are stained in blue); (**B**) The evaluation of autophagic vacuoles using MDC/PI double staining. Thus, more autophagic vacuoles can be observed in both TNBC cell lines transfected with miR-29b-3p inhibitor compared to the NC inhibitor group. In the MDA-MB-231 cell line, the presence of cell nuclei stained with PI can be observed, suggesting the activation of apoptotic processes to a late phase or necrosis. (**C**) The evaluation of apoptotic cells was evaluated by using Annexin V-FITC double staining. Post-transfection with miR-29b-3p inhibitor, apoptotic cells in both TNBC cell lines are significantly increased, suggesting that cells undergo apoptosis. An increased number of cells were found in the early and late phases of apoptosis and the necrotic phases. Images were visualized under the inverted fluorescent microscope, IX71 Olympus (20X magnification). Data were analyzed with GraphPad Prism 8 software, using Student’s *t*-test (* *p* < 0.05, ** *p* < 0.01, **** *p* < 0.0001) based on manual counting of the cells.

Forty-eight hours post-transfection, mitochondrial activity was evaluated under the fluorescence microscope. A significantly decreased mitochondrial activity was observed in both transfected TNBC cell lines compared to the NC inhibitor (data presented as % of NC inhibitor group) (Figure 3A). The nucleus fragmentation highlighted by Hoechst staining is significantly increased in transfected TNBC cells compared to the NC group. Further, autophagy was evaluated through MDC (monodansylcadaverine) and PI (propidium iodide) staining and visualized under the fluorescence microscope. Autophagy was activated for the transfected cells at a higher degree than the NC group, as shown in Figure 3B. Accumulation of MDC in the autophagy vacuoles was observed through increased dot-like structures, which give the cells a blue faded color (Figure 3B).

The inhibition of miR-29b-3p reduced the number of viable cells and altered the morphological traits of the transfected TNBC cells, suggesting that these cells underwent apoptosis. Annexin-V FITC (Fluorescein-5-isothiocyanate) and PI (propidium iodide) staining were used to confirm if apoptotic processes are activated. Increased staining for Annexin-V FITC was shown, meaning that apoptotic processes were activated in the cells transfected with the miR-29b-3p inhibitor. Meanwhile, PI staining suggests the presence of cells found in the late phase of apoptosis or necrosis (Figure 3C).

### 2.3. Identification of Differentially Expressed miRNAs as an Effect of miR-29b-3p Inhibition on TNBC Cell Lines Using Microarray Technology

**Figure 4:**
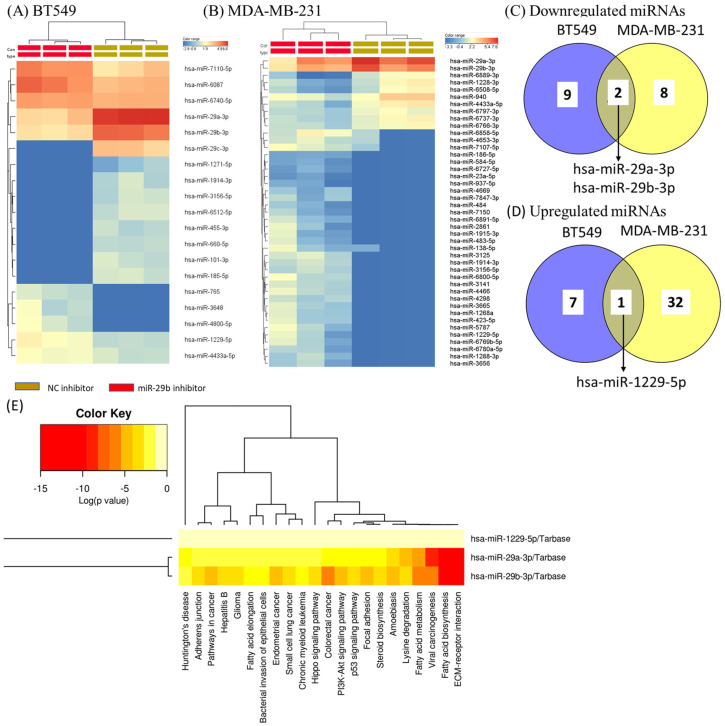
The altered miRNA pattern in TNBC cell lines as an effect of miR-29b-3p inhibition versus NC inhibitor group. Hierarchical clustering of miRNA expression in (**A**) BT549 and (**B**) MDA-MB-231 cell lines represented as heat-map, a fold change > ±1.25 and significantly expressed *p* < 0.05. (**C**) The Venn diagram used for upregulated miRNAs and (**D**) The Venn diagram used for downregulated miRNAs as an effect of miR-29b-3p inhibitor on both TNBC cell lines, generated by using Venny software; (**E**) heatmap representation of the common up- and downregulated miRs in both TNBC cell lines by highlighted the main biological processes using *DIANA*-*miRPath software*.

Because we observed significant modifications in nuclear morphology and cellular processes, we further evaluated the miRNA alteration as the effect of miR-29b-3p inhibition in both cell lines. For comparing the experimental conditions, we considered a significant FC (fold change) of ±1.25 and a *p*-value < 0.05. Using these filtering criteria, we identified 8 upregulated and 11 downregulated miRNAs in BT-549 and 33 upregulated and 10 downregulated miRNAs in MDA-MB-231 (Table 1). The miRNAs identified in BT549 cells were differentially expressed compared to those found in the MDA-MB-231 cell line. A common and a specific miRNA signature was emphasized, the common signature being represented by two downregulated transcripts (hsa-miR-29a-3p and hsa-miR-29b-3p) and one overexpressed transcript (miR-1229) (Figure 4C,D).

miR-29a-3p and miR-29b-3p are key regulatory transcripts targeting critical genes involved in cancer [19]. Essential biological processes related to these transcripts were highlighted using DIANA-miRPath. In Figure S2, we presented the target genes associated with ECM (extracellular matrix) receptor interaction and TP53 signaling. In the case of ECM receptor interaction, 18 genes were identified as common targets between miR-29a-3p and miR-29b-3p (Figure S2A) and 15 common genes related to TP53 signaling (Figure S2C).

**Figure 5:**
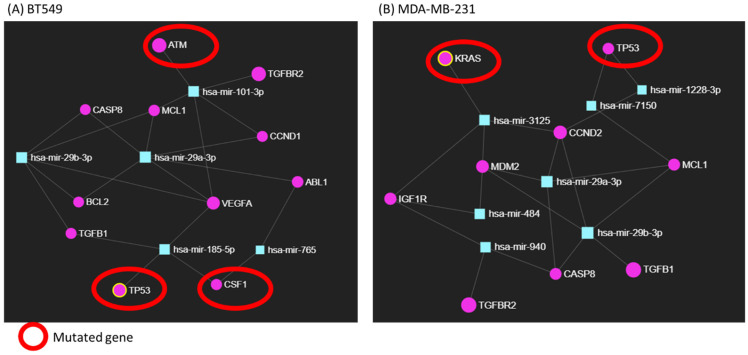
The network interaction between mRNA–miRNA is generated by using miRNet software. (**A**) BT549 cell line. (**B**) MDA-MB-231 cell line. Network generated with miRNet online tool.

**Figure 6:**
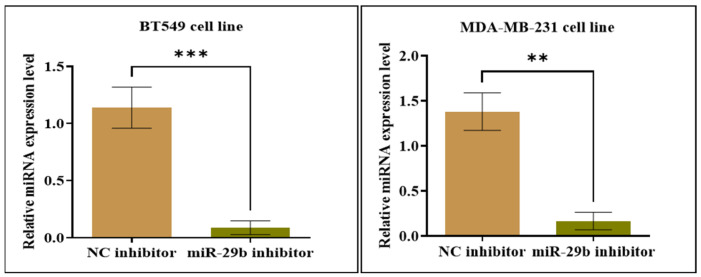
The relative expression level of miR-29b-3p in both transfected TNBC cell lines using qRT-PCR, based on ΔΔCt method (miR-29 inhibitor versus NC inhibitor group). Data were analyzed with GraphPad Prism 8 software, using Student’s *t*-test (** *p* < 0.01 and *** *p* < 0.001).

Further, by using miRNet, the interconnections established between miR-29a-3p, miR-29b-3p and targeted genes were shown (Figure 5). In this case, the same genes were also found to be mutated for each cell line. Specifically, *ATM*, *TP53* and *CSF1* were observed to be mutated in BT549 cells, while *KRAS* and *TP53* were observed to be mutated in MDA-MB-231 cells. The mutational pattern of the selected cell lines is presented in Table S1. These mutated genes modulate important biological processes, including cell proliferation, cell differentiation, cell growth, apoptosis, cell cycle arrest, DNA repair and TP53 signaling (Figure 6).

### 2.4. The miR-29b-3p Expression Level Validated in Both TNBC Cell Lines Using qRT-PCR

Further, the expression level of miR-29b-3p was validated in both TNBC cell lines post-transfection with miR inhibitor. As we can see in Figure 6, the expression profile of miR-29b-3p in both cell lines is statistically downregulated compared to the negative control (NC) inhibitor group (** *p* < 0.01; *** *p* < 0.001).

### 2.5. Evaluation by qRT-PCR of Key Genes Targeted by miR-29b-3p

**Figure 7:**
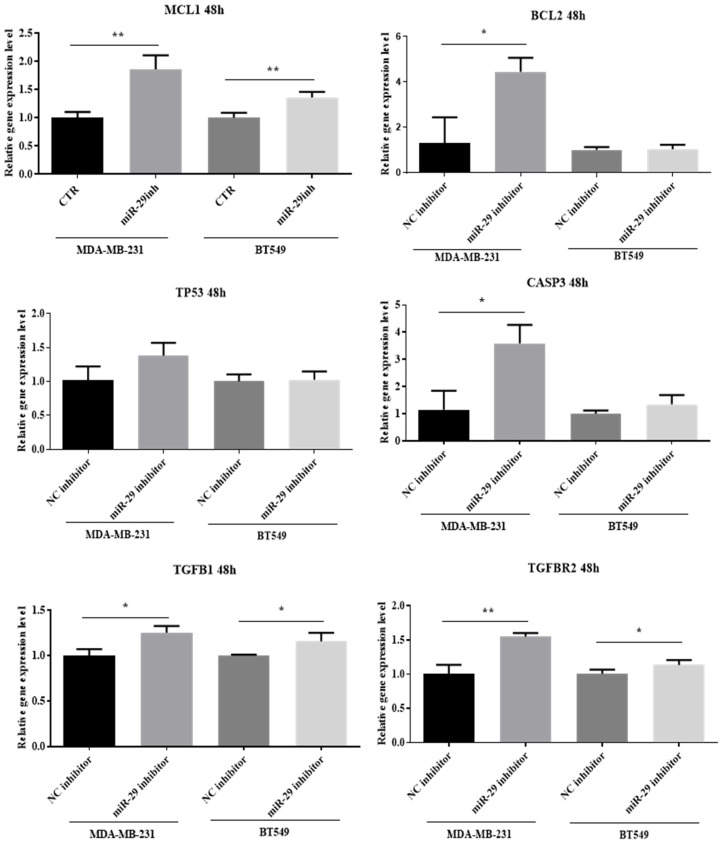
The relative expression level of selected genes according to miRNet diagram using qRT-PCR based on ΔΔCt method (miR-29 inhibitor versus NC inhibitor group). In both TNBC cell lines, BT549 and MDA-MB-231, transfected with miR-29b-3p inhibitor for 48 h, the following genes, *MCL1*, *BCL2*, *TP53*, *CASP3*, *TGFB1*, *TGFBR2,* were analyzed. Data were analyzed with GraphPad Prism 8 software, using Student’s *t*-test (* *p* < 0.05, ** *p* < 0.01).

Several genes such as *MCL1*, *BCL2*, *CASP3*, *TP53*, *TGFB1*, *TGFBR2* were analyzed in TNBC cells transfected with miR-29b-3p. As can be observed in Figure 7, the expression level of *MCL1*, *TGFB1* and *TGFBR2* is significantly upregulated in both TNBC cell lines compared to the negative control group. For *BCL2* and *CASP3,* the expression level is significantly upregulated only in the MDA-MB-231 cell line; meanwhile, the expression profile of both genes is slightly increased in the BT549 cell line but not significantly. The expression level of the *TP53* gene is slightly increased in both cell lines but not statistically significant (Figure 7).

### 2.6. Quantifying IL6 in Cell Culture Medium for Both TNBC Cell Lines Using ELISA

It is known that IL6 presents essential therapeutic relevance, being a marker associated with drug resistance mechanisms, as we observed in our previous studies [20,21,22].

**Figure 8:**
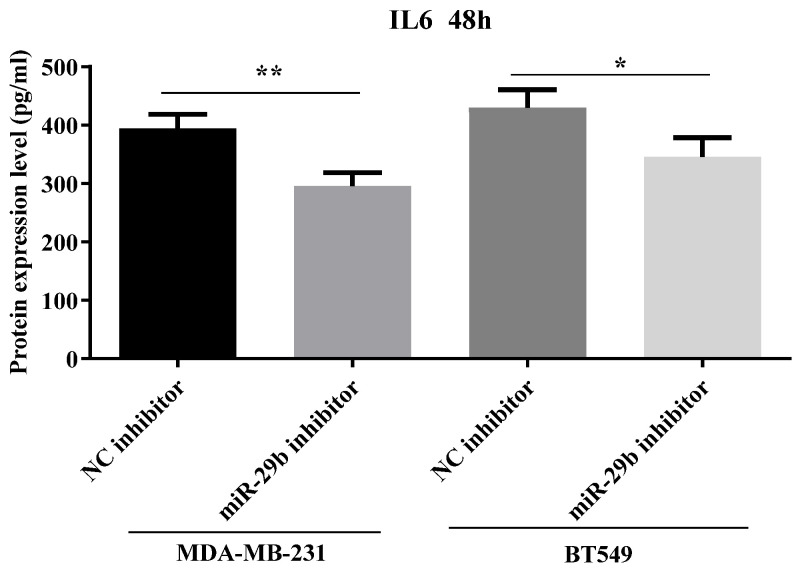
The quantification of IL6 protein in both TNBC cell lines, BT549 and MBA-MB-231, transfected with miR-29b-3p inhibitor for 48 h. Data were analyzed with GraphPad Prism 8 software, using Student’s *t*-test (* *p* < 0.05, ** *p* < 0.01).

We evaluate the IL6 protein released in the cell culture medium after transfection with NC inhibitor and miR-29b-3p inhibitor in both cell lines, BT549 and MDA-MB-231. Figure 8 shows a significant decrease in IL6 protein level in both TNBC cell lines.

## 3. Discussion

TNBC is one of the most aggressive breast cancer subtypes, presenting a high mortality rate due to the difficulty of establishing an efficient therapeutic approach. An increasing number of studies rely on miRNAs as master regulators in TNBC. miRNAs are key players in the modulation of different biological processes, including cell differentiation, cell growth, apoptosis, metastasis and drug resistance [23].

Using TCGA dataset analysis, the expression profile of miR-29b-3p was analyzed. miR-29b-3p presented a high expression profile in TNBC tissue compared to normal adjacent tissue. Then, we analyzed the expression level of miR-29b-3p in TNBC cell lines compared to the counterpart group, showing overexpression in MDA-MB-231 and BT549 cell lines compared to the normal cell line, FR2. The TNBC cell lines were further used to identify a unique molecular miRNA portrait, which can be exploited for functional studies of clinically essential miRNAs.

It has been shown that miR-29b-3p is related to breast cancer prognosis [24], including TNBC, suggesting the possibility of using miR-29b-3p as a therapeutic target in TNBC [25,26], as well as for metastatic disease [27,28]. In a previous study, the effect of the miR-29b-3p inhibitor on TNBC cell lines was investigated and revealed inhibition of cellular proliferation and colony forming ability of the MDA-MB-231 cell line [29]. In addition, it was demonstrated that miR-29b-3p promotes the progression of MDA-MB-231 cell lines by downregulating *TRAF3* and activating the NFĸB signaling pathway [29]. Herein, we used immunofluorescence to evaluate cellular morphology post-transfection, and our results showed the effect of the miR-29b-3p inhibitor on TNBC cell lines by reducing the mitochondrial activity and increasing the apoptotic cell number and autophagic vacuoles, suggesting the fact that cells undergo apoptosis. Our results in TNBC cell lines revealed a significant inhibition of cell proliferation and cell death.

The activation of *MCL1* and *BCL2* genes post-transfection with miR-29b-3p suggest the activation of drug resistance mechanisms, limiting the therapeutic effect. The implication of miR-29b-3p in drug resistance activation has also been presented in previous studies and showed the capacity to modulate the radiosensitivity in stemness cancer cells via modulating oncogenes axis [24] or via regulation of lncRNAs in endocrine-resistant breast cancer [30].

IL6 overexpression was demonstrated to be associated with resistance to therapy in multiple studies [20,31,32]. In addition, the expression level of IL6 was significantly downregulated in TNBC cell lines transfected with miR-29b-3p compared to the negative control group.

More importantly, the main implication of the *MCL1* gene in breast cancer is strongly associated with its anti-apoptotic role [33]. Therefore, the use of MCL1 selective inhibitors should be considered for developing new potential strategies for TNBC disease. Additionally, using bioinformatic tools, ECM receptor interaction and TP53 signaling were activated.

Through sequencing, some genes were observed to be mutated, such as *ATM, BRAF, TP53* and *KRAS*. These genes are intensely involved in regulating different biological processes, including DNA damage checkpoint causing cell cycle arrest, DNA repair and cell death, MAP kinase/ERK signaling pathway regulating cell division and differentiation, respectively, cell signaling pathways that regulate cell growth, maturation and apoptosis. In both cell lines, the claudin-low TNBC subtype was observed to activate oncogenic RAS signaling, mimicking the cellular plasticity. The activation of this pathway represent a critical event for the mesenchymal characteristics that define the claudin-low TNBC subtype [34].

The study aimed to investigate the functional significance of miR-29-3p and to identify molecular targets in claudin-low TNBC cells. Taken together, we determined that miR-29b-3p is an essential regulator of key pathways in TNBC. Additionally, miR-29b-3p is responsible for and involved in transcriptomic alterations, being cell-type-specific, related to the mutational pattern of each cell line. However, the precise mechanism and targets of miR-29b-3p in breast cancer and TNBC are not fully elucidated, and additional investigations are required particular to other TNBC cancer subtypes.

## 4. Materials and Methods

### 4.1. miR-29b-3p Expression Levels in TNBC

The Cancer Genome Atlas (TCGA) was used to obtain important information related to the altered miRNA pattern in human cancer, including for miR-29b-3p in TNBC and the association with overall survival rate (Table 2). The analysis was conducted using the Agilent GeneSpring GX program (Agilent Technologies, Santa Clara, CA, USA), using Benjamini–Hochberg correction and as a cut-off value, a fold change of ±1.5 and corrected *p*-value cut-off of 0.05.

### 4.2. Cell Culture

The experiments were performed using two TNBC cell lines, MDA-MB-231 and BT549. TNBC cell lines were purchased from ATCC (American Type Culture Collection, Manassas, VA, USA). MDA-MB-231 cell line was cultured in RPMI-1640 medium (Gibco, Grand Island, NY, USA), supplemented with 10% Fetal Bovine Serum (Gibco, Grand Island, NY, USA), 1% Glutamine (Gibco, Grand Island, NY, USA), 1% Penicillin-Streptomycin (Gibco, Grand Island, NY, USA) and 1% L-glutamine (Gibco, Grand Island, NY, USA). BT549 cells was cultured in RPMI-1640 medium (Gibco, Grand Island, NY, USA), supplemented with 10% Fetal Bovine Serum (Gibco, Grand Island, NY, USA), 1% Glutamine (Gibco, Grand Island, NY, USA), 1% Penicillin-Streptomycin (Gibco, Grand Island, NY, USA) and 0.023 U/mL insulin. TNBC cells were maintained at 37 °C in a humidified incubator with 5% CO_2_ atmosphere.

### 4.3. miRNA Transfection

miR-29b-3p inhibitor and negative control inhibitor were purchased from Ambion (catalogue numbers: 4,464,084 and 4,464,074, Thermo Fisher Scientific, Waltham, MA, USA). The lipofectamine 2000 (Invitrogen, Waltham, MA, USA) was used to transfect the TNBC cells according to experimental workflow presented in Figure S1. At a 50–60% confluence, TNBC cell lines were transfected with miR-29b-3p and negative control at a final concentration of 50 nM. The sequence for miR-29b-3p inhibitor is UAGCACCAUUUGAAAUCAGUGUU.

### 4.4. Cell Proliferation Assay

At a seeding density of 1 × 10^4^, cells were seeded in 96-well plate and transfected with miR-29b-3p and negative control inhibitor. Transfected cells were incubated 24 h with the transfection mix and then replaced with fresh cell culture medium for an additional 24 h. Forty-eight hours later, proliferation was assessed using MTT (Thiazolyl Blue Tetrazolium Bromide, Sigma-Aldrich, Schnelldorf, Germany) assay. Cells were incubated 2 h with 1 mg/mL MTT, and the metabolized formazan salt was resolubilized in 100 μL of DMSO (Sigma, St. Louise, MO, USA); afterwards, the absorbance was read at 570 nm with the multi-plate spectrophotometer BioTek Synergy (BioTek, Winooski, VT, USA). Each group was performed in triplicate.

### 4.5. Colony Formation Assay

At a seeding density of 1 × 10^5^, cells were plated in 12-well plate and transfected with 50 nM of miR-29b-3p inhibitor and negative control inhibitor. Forty-eight hours post-transfection, transfected cells were washed two times with Phosphate-Buffered Saline (PBS, Invitrogen, Waltham, MA, USA), tripsinized and counted. At a seeding density of 500, cells were seeded on 6-well plate and incubated at 37 °C. After 14 days, colonies were fixed in 80% methanol and stained with Trypan-blue. The result of the clonogenic assay was calculated as the number of transfected colonies with miR-29b-3p inhibitor relative to the number of negative control inhibitor colonies formed.

### 4.6. The Assessment of Mitochondrial Activity in Transfected TNBC Cell Lines

Mitochondrial activity was evaluated with Olympus IX71 inverted microscope (20X magnification) using Multi-Parameter Apoptosis Assay Kit (Cayman chemical, Ann Arbor, MI, USA; catalog no. 601280). At a seeding density of 1 × 10^4^, cells were seeded in 8-well chamber slide and transfected with 50 nM of miR-29b-3p inhibitor and negative control inhibitor for 48 h. Post-transfection, cells were stained with TMRE (Tetramethylrhodamine ethyl ester) and visualized at 560/595 nm to determine mitochondrial membrane activity potential. Additionally, cells were also stained with Hoechst and visualized in UV.

### 4.7. Apoptosis and Autophagic Vacuoles Assessment in Transfected TNBC Cell Lines

Apoptosis and autophagy were evaluated with Olympus IX71 inverted microscope using Multi-Parameter Apoptosis Assay Kit (Cayman chemical, Ann Arbor, MI, USA, cat no 600330), respectively, Autophagy/Cytotoxicity Dual Staining Kit (Cayman chemical, Ann Arbor, MI, USA, cat no. 600140) according to the manufacturer’s protocol. Cells were cultured at a seeding density of 1 × 10^4^ cells in 8-well chamber slides and transfected with 50 nM of miR-29b-3p inhibitor and negative control inhibitor for 48 h. To evaluate the apoptotic effect, cells were stained with Annexin-V FITC used to identify different types of cell death (early and late apoptosis) and evaluated at 485/535 nm. PI (propidium iodide), which is a DNA-binding dye molecule, is used to differentiate late apoptosis from necrosis being evaluated at 535/617 nm. The autophagic vacuoles were stained with monodansylcadaverine (MDC) and visualized in UV. Cellular death was evaluated at 520/610 nm using PI (propidium iodide).

### 4.8. miRNA Altered Pattern as Effect of miR-29b Transfection in TNBC Cells

The RNA from the two selected groups (triplicate, 6 well plated), miR-29b inhibitor and NC inhibitor transfected cells, was extracted using TriReagent (Invitrogen, Waltham, MA, USA) according to manufacturer’s instruction and quantified by using NanodropND2000 spectrophotometer (ThermoFisher Scientific, Waltham, MA, USA). miRNA alterations were evaluated starting from 100 ng of total RNA using microRNA Spike-In kit and the labeling miRNA Complete Labeling and Hyb Kit (Agilent technologies, Santa Clara, CA, USA); additional purification step was performed using Micro Bio-Spin 6 (Bio-Rad, Mississauga, ON, Canada) spin columns. For the 20 h at 55 °C, Agilent SurePrint Human miRNA v21.0 microarray slides (G4872A) (Agilent technologies, Santa Clara, CA, USA) were used for hybridization; then, the slides were washed and scanned using an Agilent Microarray Scanner (Agilent technologies, Santa Clara, CA, USA). The data were extracted using Agilent Feature Extraction software (Agilent Technologies, Santa Clara, CA, USA); Agilent GeneSpring GX program (Agilent Technologies, Santa Clara, CA, USA) was used to obtain differentially expression miRNAs, considering as cut-off value with a FC ± 1.25 and *p*-value ≤ 0.05.

**Genetic alteration evaluation using next-generation sequencing panel on Ion Torrent.** DNA from TNBC cell lines was extracted using the Purelink Genomic DNA mini kit (ThermoFisher Scientific, Waltham, MA, USA) following the manufacturer’s instructions. An amount of 20 ng of DNA was used for sequencing using the Ion AmpliSeq Cancer Hotspot Panel v2 (ThermoFisher Scientific, Waltham, MA, USA) and the Ion AmpliSeq Library 2.0 kit (ThermoFisher Scientific, Waltham, MA, USA) using the Ion Torrent Personal Genome Machine (ThermoFisher Scientific, Waltham, MA, USA) [20].

### 4.9. Gene and miRNA Expression Evaluation Using qRT-PCR

At a seeding density of 5 × 10^5^, cells were seeded in 6-well plate and transfected with miR-29b-3p inhibitor and negative control inhibitor. Forty-eight hours post-transfection, cells were collected and total RNA was extracted using TriReagent (Ambion, Austin, TX, USA) according to manufacturer’s instruction. RNA concentration was quantified by using a NanoDrop 2000 spectrophotometer (ThermoFisher Scientific, Waltham, MA, USA).

An amount of 1000 ng of total RNA was reverse-transcribed into cDNA using High-Capacity cDNA Reverse Transcription Kit (Applied Biosystems, Waltham, MA, USA). Gene expression evaluation was conducted using SYBR Select Master Mix (Applied Biosystems, Waltham, MA, USA) and RT-qPCR was performed on ViiA^™^7 System (Applied Biosystems, Waltham, MA, USA) using a 384-well plate. As an internal control, we used B2M, the primer sequences being presented in Table 3.

An amount of 50 ng of total RNA was reverse-transcribed into cDNA using TaqMan MicroRNA Reverse Transcription Kit (Applied Biosystems, Waltham, MA, USA). miRNA evaluation was conducted using TaqMan Master Mix (Applied Biosystems, Waltham, MA, USA) and RT-qPCR 4 was performed on ViiA^™^7 System (Applied Biosystems, Waltham, MA, USA) using a 384-well plate. We used RNU48 (Applied Biosystems, Waltham, MA, USA cat no. 001006) as an internal control and a specific primer for miR-29b-3p (Applied Biosystems, Waltham, MA, USA cat no. 000413).

Relative quantification for gene and miRNA was conducted using the 2-ΔΔCT method [35], according to the recommendation of Vandesompele J for selecting the most relevant housekeeping genes and internal controls [36].

### 4.10. IL6 Quantification from the Cell Culture Medium

Forty hours post-transfection, cell culture medium from both TNBC cell lines, MDA-MB-231 and BT549, was collected. The expression level of IL6 released in the cell culture medium was detected through ELISA using the IL6 DuoSet ELISA (R&D System, Minneapolis, MN, USA, cat no. DY206) according to the manufacturer’s instructions.

### 4.11. Statistical Analysis

Statistical analyses were carried out using GraphPad Prism version 8 software, miRNet Field [36], Venny 2.1.0 (https://bioinfogp.cnb.csic.es/tools/venny/, accessed on 24 June 2022), DIANA-miRPath Field [37]. Resulted data were expressed as mean ± SD (standard deviation). The difference between experimental conditions and controls was analyzed using a t-test (statistically significant was considered *p* < 0.05).

## 5. Conclusions

According to our data, miR-29b-3p in MDA-MB-231 and BT549 cell lines is overexpressed. By evaluating cellular and molecular processes after transfection with miR-29b-3p, the effect was strongly associated with the inhibition of cell proliferation, as well as with the activation of apoptotic processes and autophagic vacuoles. More importantly, the overexpression of MCL1 and BCL2 genes, suggesting the implication in activating drug resistance mechanisms, might limit the therapeutic implication. To improve the therapy for TNBC patients, further investigations are needed to be tested by combining the miR-29b-3p inhibitor with different therapeutic agents to counteract drug resistance mechanisms.

## Figures and Tables

**Table 1:** The altered miRNAs pattern on BT549 and MDA-MB-231 cell lines as an effect of miR-29b-3p inhibition.

BT549			MDA-MB-231		
Systematic_Name	FC	Corr *p* Value	Systematic_Name	FC	Corr *p* Value
hsa-miR-29c-3p	−96.24	0.0001	hsa-miR-6889-3p	−15.41	0.011
hsa-miR-185-5p	−16.09	0.0002	hsa-miR-6508-5p	−6.93	0.025
hsa-miR-6512-5p	−15.81	0.0003	hsa-miR-1228-3p	−6.12	0.036
hsa-miR-101-3p	−14.33	0.0004	hsa-miR-4433a-5p	−5.19	0.013
hsa-miR-3156-5p	−13.59	0.0008	hsa-miR-29a-3p	−5.01	0.045
hsa-miR-660-5p	−11.97	0.0000	hsa-miR-29b-3p	−3.62	0.043
hsa-miR-455-3p	−11.63	0.0003	hsa-miR-6737-3p	−3.37	0.006
hsa-miR-1914-3p	−11.62	0.0020	hsa-miR-940	−3.10	0.047
hsa-miR-29a-3p	−11.24	0.0016	hsa-miR-6797-3p	−2.85	0.002
hsa-miR-29b-3p	−7.17	0.0004	hsa-miR-6766-3p	−2.69	0.009
hsa-miR-1271-5p	−5.83	0.0284	hsa-miR-6800-5p	19.82	0.000
hsa-miR-4800-5p	19.97	0.0044	hsa-miR-3125	19.51	0.000
hsa-miR-3648	16.12	0.0091	hsa-miR-4466	17.33	0.000
hsa-miR-765	14.67	0.0004	hsa-miR-1914-3p	15.88	0.000
hsa-miR-7110-5p	3.74	0.0433	hsa-miR-3141	15.33	0.000
hsa-miR-1229-5p	2.96	0.0060	hsa-miR-5787	14.21	0.004
hsa-miR-6087	2.79	0.0478	hsa-miR-6858-5p	14.05	0.024
hsa-miR-4433a-5p	2.12	0.0433	hsa-miR-3665	13.60	0.001
hsa-miR-6740-5p	1.36	0.0284	hsa-miR-423-5p	13.54	0.001
			hsa-miR-3156-5p	13.54	0.000
			hsa-miR-4298	12.98	0.000
			hsa-miR-7107-5p	12.87	0.016
			hsa-miR-1268a	12.83	0.001
			hsa-miR-6769b-5p	10.73	0.006
			hsa-miR-1229-5p	10.08	0.018
			hsa-miR-1288-3p	9.33	0.001
			hsa-miR-6891-5p	8.77	0.004
			hsa-miR-4653-3p	8.51	0.042
			hsa-miR-2861	8.15	0.035
			hsa-miR-7847-3p	8.01	0.002
			hsa-miR-483-5p	7.97	0.014
			hsa-miR-6780a-5p	7.70	0.006
			hsa-miR-3656	7.55	0.004
			hsa-miR-1915-3p	7.09	0.028
			hsa-miR-138-5p	6.71	0.042
			hsa-miR-4669	6.43	0.001
			hsa-miR-7150	5.09	0.018
			hsa-miR-484	4.49	0.017
			hsa-miR-186-5p	3.45	0.000
			hsa-miR-6727-5p	3.14	0.006
			hsa-miR-584-5p	3.09	0.004
			hsa-miR-937-5p	2.71	0.048
			hsa-miR-23a-5p	2.11	0.023

**Table 2:** Clinical data for TNBC patients using TCGA Data.

Demographics	TNBC
No. of cases	
Females	112
Age	
Median, Range	54 (29–90)
TNM	
T1	27
T2	70
T3	11
T4	4
Tx	-
N0	72
N1	25
N2	11
N3	4
Nx	-
M0	95
Mx	17
Tumor grade (I–IV)	
I:	20
II:	70
III:	18
IV:	1
Unknown:	3

T: tumor; N: node; M: metastasis.

**Table 3:** Primer sequences used for gene expression evaluation.

Primer	Sequence
MCL1	FW-TGTCCAGTTCCGAAGCAT/ RV-AAGCGAATGGGCAGGTCGT
BCL2	FW-GCGCTACAGTTCCACAAAGG/ RV-AGTACCTGAACCGGCACCT
TP53	FW: CCC TTT TTG GAC TTC AGG TG/ RV: AGG CCT TGG AAC TCA AGG AT
Caspase 3	FW-GCTTGTCGGCATACTGTTTCAG/ RV-AGAACTGGACTGTGGCATTGAG
TGFβ1	FW-ACTACTACGCCAAGGAGGTCAC/ RV-TGCTTGAACTTGTCATAGATTTCG
TGFΒR2	FW-CACCGCACGTTCAGAAGTC/ RV-TGGATGGGCAGTCCTATTACA
B2M	FW-CACCCCCACTGAAAAAGATGAG/ RV-CCTCCATGATGCTGCTTACATG

## Data Availability

Available on request to the corresponding author.

## Supplementary Materials

The following supporting information can be downloaded at: https://www.mdpi.com/article/10.3390/ijms24055048/s1.
