# Supplementary material for: Discovering the Biological Significance and Therapeutic Potential of miR-29b-3p in Triple-Negative Breast Cancer
Source: Int J Mol Sci. 2023 Mar 6;24(5):5048. doi: 10.3390/ijms24055048 (PMC10003717; doi:10.3390/ijms24055048)
Supplement: Supplementary file 1 [file ijms-24-05048-s001.zip › ijms-2157762-supplementary.pdf]

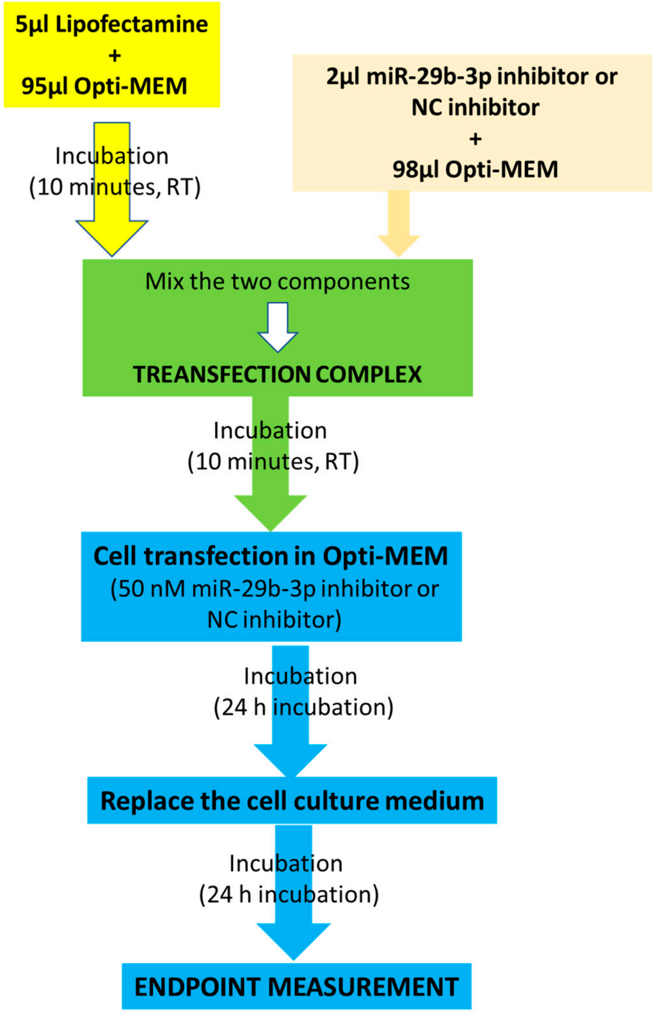

Figure S1. Experimental workflow for transfection procedure. TNBC cell lines were transfected with miR-29b-3p and negative control (NC) inhibitor at a final concentration of 50 nM and collected 48 hours post transfection. .

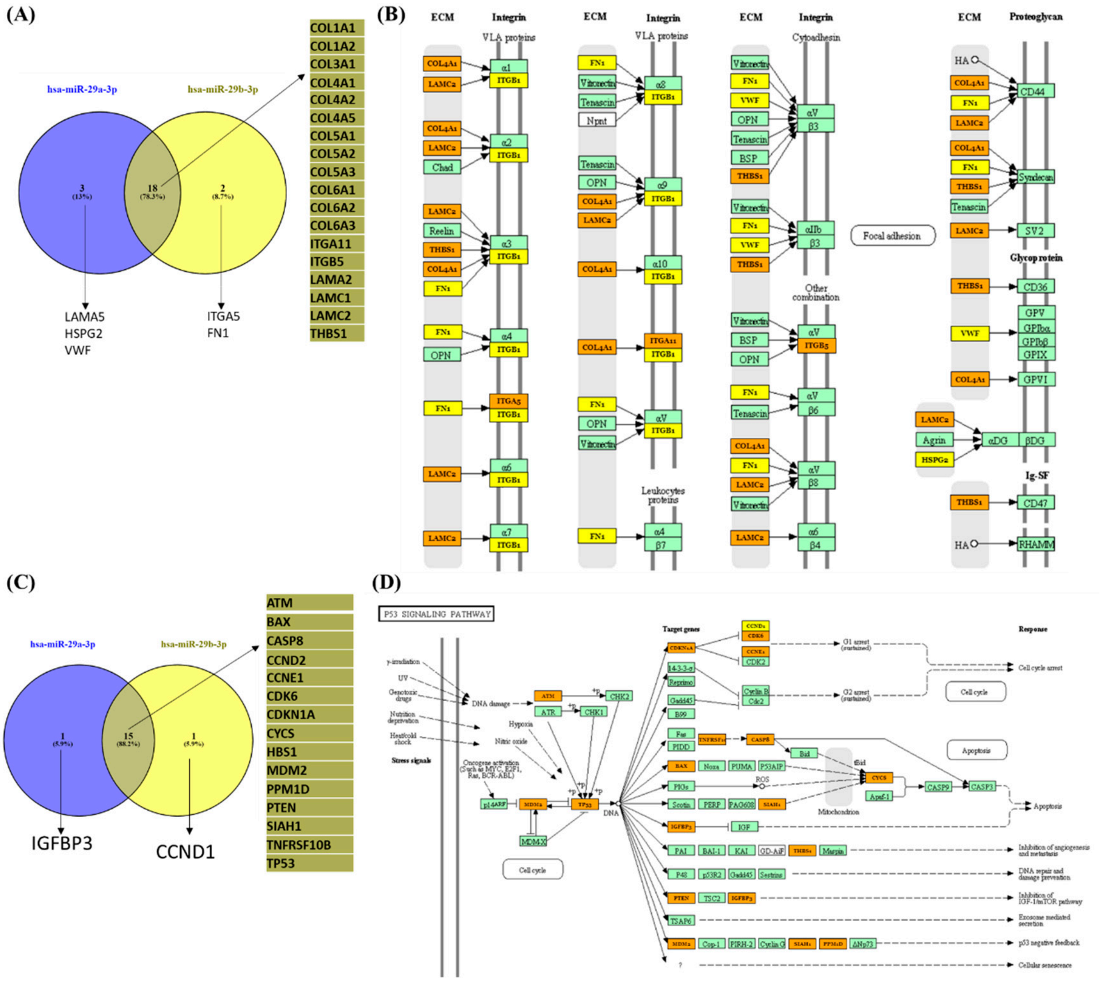

Figure S2. The main genes targeted by miR-29a-3p and miR-29b-3p identified using DIANA-miRPath v3.0. (A) The Venn diagram used to highlight the common genes between miR-29a-3p and mir-29b-3p related to ECM receptor interaction. **(B)** ECM receptor interaction network. **(C)** The Venn diagram used to highlight the common genes between miR-29a-3p and mir-29b-3p related to TP53 signaling. **(D)** TP53 signaling network. The targeted genes are highlighted in yellow (gene targeted by 1 selected miRNA from the list), orange (gene targeted by >1 selected miRNAs) and red (gene from the network that is not targeted by selected miRNAs).

Table S1. Mutational pattern for untreated BT549 and MDA-MB-231 cell lines (the NGS data were align to hg19)

Sample	**Locus**	**Genes**	Allele Frequency %	**Coding**	Amino Acid Change	**ClinVar**
MDA-MB-231	chr3:178917005	PIK3CA	33.38	c.352+40A>G		VUS
	chr4:55980239	KDR	30.88	c.798+54G>A		VUS
	chr7:140481417	BRAF	55.55	c.1391G>T	p.Gly464Val	Pathogenic/Likely pathogenic
	chr12:25398280	KRAS	60.52	c.38G>A	p.Gly13Asp	Pathogenic
	chr17:7577094	TP53	99.3	c.839G>A	p.Arg280Lys	Pathogenic/Likely pathogenic
	chr17:7579472	TP53	96.63	c.215C>G	p.Pro72Arg	Benign
	chr18:48586344	SMAD4	56.27	c.955+58C>T		VUS
BT549	chr2:212812097	ERBB4	100	c.421+58A>G		VUS
	chr5:149433595	CSF1R	32.28	c.*37AC>C		VUS
	chr5:149433597	CSF1R	32.22	c.*35C>TC		VUS
	chr11:108236264	ATM	32.67	c.*29C>G		Likely benign
	chr13:28610183	FLT3	100	c.1310-3T>C		VUS
	chr17:7577528	TP53	99.94	c.747G>C	p.Arg249Ser	VUS
	chr17:7577533	TP53	100	c.747delG	p.Arg249SerfsTer96	VUS
